# From Biodiversity to Biocompatibility: Insights from the Special Issue “Analysis and Biological Evaluation of Bioactive Compounds from Natural Sources—Second Edition”

**DOI:** 10.3390/molecules31101678

**Published:** 2026-05-15

**Authors:** Rosanna Avola, Luana Pulvirenti

**Affiliations:** 1 Department of Medicine and Surgery, Kore University Enna, 94100 Enna, Italy; 2 Istituto di Chimica Biomolecolare del CNR, Via P. Gaifami, 18, 95126 Catania, Italy

## 1. Introduction

Contemporary research in food chemistry, pharmacognosy, and translational sciences is undergoing a profound methodological convergence. The focus is shifting from the simple identification of bioactive molecules toward a systemic understanding of how genotype, environment, technological processes, and biological matrices interact to determine the efficacy of functional ingredients. The Special Issue “*Analysis and Biological Evaluation of Bioactive Compounds from Natural Sources—Second Edition*” brings together eight seemingly heterogeneous contributions—ranging from minor cereals to brown algae, and from wild mushrooms to lipid formulations for blood preservation—that offer a coherent overview of an ongoing epistemological transition: the shift from a reductionist approaches toward an integrated scientific framework, where complexity represents a design parameter rather than a limitation.

This collection is part of a structured editorial process, initiated with the previous editorial of the first edition of the Special Issue [1], which defined the methodological framework for an integrated approach between analytical characterization and biological evaluation of natural compounds. This work represents a further development of this paradigm, demonstrating how the convergence of different disciplines can translate into concrete strategies for functional enhancement.

In this editorial, we propose a thematic reinterpretation of these works, organizing them into four conceptual axes: (i) chemotypic stability as a prerequisite for industrial reproducibility; (ii) process engineering as a lever for bioactive modulation; (iii) mechanistic selectivity as a criterion for translational safety; and (iv) systemic biocompatibility as a bridge between nutrition and clinical applications.

## 2. Chemotypic Stability: When Variability Becomes a Design Parameter

The first conceptual pillar that emerges from a cross-reading of these studies is the recognition that the bioactive profile of a plant matrix is not a static attribute, but the dynamic result of Genotype × Environment × Process (G × E × P) interactions. Ignoring this complexity risks transforming functional potential into uncontrolled variability.

The study on rye (*Secale cereale* L.) exemplarily demonstrates how the content of dietary fiber and alkylresorcinols—compounds key to glycemic regulation and microbiota health—is driven predominantly by environmental conditions and G × E interactions, with the contribution of genotype alone accounting for less than 11% (Contribution 1). This finding aligns with recent evidence showing that environmental factors influence cereal crop quality traits to a greater extent than the genotype itself [2], confirming that selection for chemotypic stability requires multi-environmental approaches and predictive models such as AMMI to identify resilient lines [3,4]. Far from being a limitation, this variability constitutes a methodological warning: to develop rye products with predictable nutritional profiles, it is not enough to identify the “richest” genotype, but it is necessary to select chemically stable lines. The AMMI analysis identifies the KWS Serafino hybrid as a model of chemostability, confirming that contemporary breeding must explicitly integrate bioactive profile stability as a primary phenotypic trait, especially in scenarios of increasing climate variability [5].

A similar lesson emerges from the characterization of 49 spontaneous species of the Bulgarian flora, where the extraordinary variability in glyceride oil content (0.5–40.6%) and tocopherol profile (4.4–8066.5 mg/kg) responds to adaptive and ecological logics rather than rigid taxonomic hierarchies (Contribution 2). Species such as *Allium sardoum*, *Verbascum lychnitis* and *Broussonetia papyrifera* emerge as promising candidates, but this screening raises a crucial operational question: biodiversity alone is not enough; a functional mapping is needed to guide their sustainable valorization. An exemplary approach in this sense is illustrated by the integrated study on wild Crocus species, where the systematic correlation between phenotypic traits and phytochemical profile allows the identification of promising chemotypes for nutraceutical applications [6]. Recent work on chemotypic variability in genera such as *Silybum* demonstrates that characterization at the single-plant level is essential to identify stable chemotypes, since apparently homogeneous populations can harbor distinct metabolic profiles [7,8]. Botanical taxonomy does not predict the chemical profile; a data-driven functional approach that combines chemotyping, analytical validation, and predictive models is needed to turn the “natural case” into a “designed ingredient” [9,10]. The study on the protein fraction isolated from *Prunus padus* L. flour also confirms this perspective: the alkaline extraction process not only enriched the protein content (from 15.44 to 39.72 g/100 g), but also selectively modulated the profile of bioactive compounds, reducing cyanogenic amygdalin by 90% while preserving polyphenols and antioxidant activity (Contribution 3). This result fits into an emerging trend of valorization of underutilized plant resources and agro-industrial by-products, where the optimization of extraction protocols becomes an integral part of the definition of the safety and functionality profile [11,12]. Recent studies highlight that the stability of phytochemicals during processing is an essential prerequisite for the development of functional foods: promising compounds in vitro lose efficacy if degraded during extraction, formulation or storage [9,10]. Functionality is not passively inherited; it is actively selected—and the extraction process becomes an integral part of defining the security profile.

## 3. Process Engineering: Technology as a Multiplier of Bioactive Value

If chemotypic stability is the prerequisite, process optimization is the operational lever for transforming potential into real benefit. Three contributions clearly illustrate how seemingly marginal technological choices can decisively shape the final product profile, a concept widely supported by recent studies on advanced food processing [13,14].

In the case of freeze-dried snacks obtained from *Lactarius deliciosus* caps fermented with probiotic strains, the choice of pretreatment (boiling in water vs. microwave) significantly determines the profile of minerals, phenolic compounds, lovastatin, and indoles (Contribution 4). Microwaves better preserve potassium, selenium, iron, manganese, and antioxidant activity, also promoting the survival of lactobacilli; water, on the other hand, concentrates lovastatin and 6-methyl-D, L-tryptophan. This result is consistent with recent studies demonstrating that microwave treatment, thanks to selective dielectric heating, minimizes the thermal degradation of thermolabile compounds and preserves the structural integrity of biological matrices [15,16]. However, the most interesting result lies in microbiological stability: after six months of storage, only the microwave-treated product fermented with *L. plantarum* SWA016 maintains high probiotic counts (>7 log CFU/g). The literature confirms that probiotic survival during storage critically depends on the synergy between matrix pre-treatment, strain selection, and stabilization technologies such as freeze-drying with cryoprotectants [17,18]. Functionality is not only designed during the formulation phase; it is preserved during storage.

A similar approach guides the study on the enrichment of beer wort with hemp seeds (*Cannabis sativa* L.): the controlled malting of the seeds selectively modulates the release of thiamine and riboflavin, while the use of unmalted seeds maximizes the extraction of phenolic acids and quercetin (Contribution 5). Crucially, the addition of hemp does not compromise critical technological parameters (saccharification times < 10 min and improved filtration), demonstrating that functional innovation and industrial feasibility can coexist. This approach aligns with recent “functional brewing” strategies, where the integration of botanical ingredients requires a balance between bioactive extraction and the maintenance of the rheological and fermentative properties of the wort [19,20]. Recent studies also highlight that malt can activate endogenous enzymes that favor the bioavailability of micronutrients, offering a further degree of freedom for the design of functional beers [21].

The logic of “circular valorization” also emerges strongly in studies on waste matrices: *Salvia officinalis* hydrodistillation waters, for example, retain a residual phenolic profile with significant anti-inflammatory activity in vitro [22], confirming that primary extraction processes do not necessarily exhaust the bioactive potential of a matrix. Similarly, the use of small-sized tomato pomegranates as an ingredient for functional bread demonstrates that standardizing processing protocols can convert an agro-industrial by-product into a stable and reproducible nutraceutical resource [23]. Finally, the study on flavonoids isolated from lemon juice waste, characterized using multispectroscopic and in silico approaches, illustrates how extraction process optimization and mechanistic validation must proceed in parallel to transform a residue into a functional ingredient with a defined target [24].

Finally, the study on sodium alginates extracted from *Ecklonia radiata* and *Sargassum elegans* highlights how finely tuned structural parameters—molecular weight, M/G ratio, and degree of branching—significantly influence the inhibitory activity towards digestive enzymes (Contribution 6). The comparison with a commercial alginate—which, despite having similar viscosity, shows negligible activity—demonstrates that the biological efficacy does not depend on the stoichiometry of the components, but on hidden variables related to the extraction and purification process. Recent studies on marine polysaccharides confirm that the structure–function relationship is highly specific: small variations in the M/G ratio or in the molecular weight can drastically alter the affinity for enzymatic sites or the ability to form viscoelastic complexes [25,26]. This underlines the need to standardize the chemical–physical characterization protocols to allow meaningful comparisons between studies and industrial reproducibility [27].

## 4. Mechanistic Selectivity: From “What” to “How” in the Design of Functional Ingredients

A third conceptual axis unifying these contributions is the shift from a purely phenomenological assessment of biological activity to a thorough mechanistic characterization. This evolution is essential for distinguishing specific effects from experimental artifacts and for designing rational combination strategies, a key principle of precision nutrition and network pharmacology [28,29].

The review of artemisinin derivatives in colorectal cancer represents a model in this regard: rather than listing fragmented mechanisms, the authors construct a “multidimensional attack network” in which ADs act simultaneously to eradicate cancer stem cells, reactivate apoptosis and induce ferroptosis, suppress pro-survival pathways, and remodel the immune microenvironment (Contribution 7). This conceptual framework positions ADs not as simple cytotoxic agents, but as “multidimensional modulators” capable of enhancing conventional chemotherapy by acting as sensitizers. Recent studies on ferroptosis confirm that the selective induction of this form of cell death represents a promising strategy to overcome resistance to conventional drugs, especially in tumors with impaired iron metabolism [30,31]. Furthermore, the integration of network pharmacology approaches and multi-omics analysis is becoming essential to decipher the complex interactions between phytocompounds and cellular pathways, accelerating the clinical translation of natural candidates [32,33].

The importance of a thorough mechanistic characterization is further underlined by studies integrating computational and experimental approaches: the isolation and validation of pancreatic lipase-inhibiting flavonoids from lemon waste, supported by molecular docking and kinetic analysis [24], represents a model for distinguishing specific interactions from nonspecific effects. Similarly, reviews of complex therapeutic targets—such as the role of molecular chaperones in lysosomal storage diseases such as Krabbe disease—highlight how understanding cellular pathways is essential for designing rational intervention strategies and identifying novel therapeutic candidates of natural origin [34].

Similarly, the study on alginates not only confirms a generic inhibitory activity, but also demonstrates a crucial functional selectivity: significant inhibition of α-glucosidase (~40%) and maltase (~30%), with negligible effects on α-amylase and sucrase (Contribution 6). This selectivity is physiologically relevant: by targeting the terminal phases of carbohydrate digestion, alginates could modulate postprandial glycemic peaks without interfering with the initial digestion of starch, potentially reducing the gastrointestinal side effects associated with broad-spectrum inhibitors such as acarbose [35,36]. Fluorescent quenching and kinetic analysis confirm a direct and specific interaction, strengthening the hypothesis of a structural rather than aspecific mechanism. Recent studies on enzymatic inhibition by marine polysaccharides suggest that binding specificity depends on the three-dimensional conformation and the distribution of charged functional groups, paving the way for rational engineering strategies of biopolymers for nutraceutical applications [37,38].

## 5. Systemic Biocompatibility: When Nutrition Meets Clinical Conservation

The final thematic axis concerns the ability of some natural preparations to act simultaneously on multiple physiological fronts, opening up application scenarios that transcend the traditional distinction between food, supplement, and medical device. This “multi-target” approach is increasingly recognized as essential for addressing the complexity of human biological systems [39,40].

The study on the preparation based on extra virgin olive oil and *Mentha piperita* essential oil (ATP-oil^®^) clearly illustrates this potential: in serum-free culture conditions, the treatment prolongs erythrocyte viability (35–45% at day 18 vs. rapid decline in controls) and simultaneously limits background bacterial proliferation (Contribution 8). The rationale rests on two synergistic pillars: the antioxidant and membrane-protective properties of olive polyphenols, and the broad-spectrum antimicrobial activity of mint terpenes. Recent studies confirm that extra virgin olive oil polyphenols, particularly hydroxytyrosol and oleuropein, protect erythrocyte membranes from lipid peroxidation and eryptosis by modulating redox pathways and stabilizing membrane fluidity [41,42]. In parallel, essential oils from Lamiaceae, rich in menthol and 1,8-cineole, exert antimicrobial activity by destabilizing bacterial membranes and interfering with ionic transport systems [43,44]. Particularly intriguing is the positive correlation between oral intake and in vitro erythrocyte survival, which suggests a possible systemic “priming” effect: recent studies on “nutritional priming” demonstrate that previous exposure to bioactive compounds can modulate gene expression and membrane lipid composition, increasing cellular resilience to subsequent stress [45,46].

In parallel, the evaluation of anti-inflammatory activity using *in vitro* models—such as in the case of extracts from sage processing water [22]—provides essential preliminary data to validate the safe use of complex preparations in clinical or nutraceutical contexts.

However, the study also highlights a methodological warning: the “mimicking” laboratory preparation, while replicating the same volume ratios, does not reproduce the protective effect of the commercial product. This gap demonstrates that biological efficacy depends on hidden variables—degree of refinement, oxidative profile, and homogenization techniques—which require standardization and rigorous quality control before any clinical translation. The literature on complex natural products highlights that the reproducibility of biological effects requires not only the standardization of the marker’s active principles, but also the characterization of the lipid matrix, the oxidative profile, and the stability over time [47,48]. Furthermore, the absence of quantitative evaluations of the bacterial load (CFU/mL) and of in-depth mechanistic assays (lipidomics and proteomics) represents a limitation that future research will have to overcome to fully validate the translational potential of this approach [49].

## 6. Discussion

A comprehensive reading of these contributions highlights a rapidly evolving scientific paradigm. It is no longer a matter of isolating molecules and testing their efficacy in simplified systems, but of actively managing the entire bioactive value chain, from the field to the physiological target. G × E × P variability emerges not as background noise to be eliminated, but as an information vector to be modeled. Chemometric approaches, AMMI models, and machine learning techniques applied to the food matrix are becoming indispensable tools for anticipating environmental fluctuations and ensuring industrial reproducibility. At the same time, translational stability is emerging as the new gold standard: identifying a promising compound is only the first step, while the real challenge lies in preserving its structural integrity, bioavailability, and efficacy after processing, packaging, and its shelf life. The ability to maintain high probiotic counts after six months, to preserve the chemostability of rye fiber, or to maintain enzymatic selectivity in alginates is not simply analytical data, but an indicator of technological maturity.

Biodiversity, if not accompanied by rigorous functional mapping, risks remaining an unexplored reservoir. Botanical taxonomy or geographic origin are not predictive of the chemical profile; a data-driven approach that integrates chemotyping, multivariate analytical validation, and predictive models is needed. Likewise, technological choice is not neutral: microwave versus water, malting versus direct use, one probiotic strain versus another, and freeze-drying parameters; every decision actively modulates the nutritional profile. Processes must be tailored to the target bioactives, not vice versa. Finally, in-depth mechanistic characterization—enzyme kinetics, fluorescent quenching, network pharmacology, and multi-omics analysis—represents the essential filter for distinguishing specific effects from experimental artifacts and for designing rational combinations for precision nutrition.

## 7. Conclusions

The contributions presented in this Special Issue collectively demonstrate that the functional enhancement of natural resources extends beyond the identification of single bioactive molecules. Rigorous in their methods, innovative in their concepts, and attentive to practical implications, these studies demonstrate that biological complexity is not an obstacle to industrial standardization, but the very foundation of rational design. The convergence of agronomy, food technology, microbiology, analytical chemistry, and translational sciences enables a shift beyond the reductionist logic of the single active compound, embracing a systemic vision in which genotype, environment, process, and biological matrix interact continuously. The challenge for the coming years will be to translate this integrated understanding into standardized, validated, and scalable protocols, placing both scientific excellence and tangible impact on human health and environmental sustainability at the center.

## 8. Prospective Future

The path toward the full functional exploitation of natural resources will require a methodological and organizational leap. First, it will be necessary to develop predictive platforms capable of integrating climatic, genomic, and process data to simulate chemotypic stability in silico and optimize transformation pathways even before field or plant testing. Second, the standardization of complex preparations will have to evolve from the simple quantification of active markers towards holistic profiling that includes lipid matrix, oxidative state, structural conformation of biopolymers, and synergistic interactions between components—a principle already highlighted in the editorial that inaugurated this Special Issue [1]. Collaboration between research institutions, regulatory authorities, and industry will be crucial to define shared validation protocols capable of bridging the gap between in vitro results and clinical or food application. Finally, the integration of multi-omics approaches, artificial intelligence, and biological simulation models will allow us to move from generic functional nutrition to personalized strategies, in which the food matrix or natural preparation is modulated based on the subject’s metabolic phenotype, intestinal microbiota, and health status. Only through this disciplinary and methodological convergence will we be able to transform the richness of plant and marine biodiversity into a new generation of scientifically validated, accessible, and sustainable health solutions.
